# Semi-field assessment of the Gravid *Aedes* Trap (GAT) with the aim of controlling *Aedes* (*Stegomyia*) *aegypti* populations

**DOI:** 10.1371/journal.pone.0250893

**Published:** 2021-04-29

**Authors:** Alvaro E. Eiras, Laila H. Costa, Luciane G. Batista-Pereira, Kelly S. Paixão, Elis P. A. Batista

**Affiliations:** Laboratory of Innovation Technologies in Vector Control, Department of Parasitology, Biological Sciences Institute, Federal University of Minas Gerais, Belo Horizonte, Brazil; Universidade Federal do Rio de Janeiro, BRAZIL

## Abstract

The mosquito *Aedes aegypti* is the main vector of arboviroses and current approaches to control this vector are not sufficiently effective. Adult traps, such as the BG-Sentinel (BGS), have been successfully used for mosquito surveillance and can also suppress vector populations. A new “passive” trap for gravid *Ae*. *aegypti* (Gravid *Aedes* Trap—GAT) has been shown efficient for *Aedes* collection and suppress *Ae*. *albopictus* populations using mass trapping techniques. Here the GAT was evaluated for the first time as a new tool to control *Ae*. *aegypti* in semi-field conditions using simulated outdoor environments (SOE). Two identical large screened chambers inside of a SOE containing different numbers and sizes of artificial breeding sites were used to assess the trapping efficiency of the GAT. One hundred mosquitoes were released into the chambers, and recapture rates evaluated after 48h. The parity status of the captured mosquitoes was also recorded. The number of eggs laid, and breeding productivity were also monitored when using different numbers and sizes of breeding sites. The BGS trap was used here as a control (gold standard) trap to compare capture rates to those of the GAT. The GAT recaptured between 50–65% of the mosquitoes independent of the number and sizes of the breeding sites in the SOEs, whereas the BGS recaptured 60–82% of the females. Both traps showed similar results regarding to the parity status of recaptured mosquitoes. Our results confirmed the effectiveness of GAT for the capture of adult female *Ae*. *aegypti* in simulated field environments. The BGS trap recaptured gravid *Ae*. *aegypti* before egg-laying in different sizes and number of breading sites, whereas the oviposition activity occurred prior to recapture mosquitoes in the GAT. Based on the results, we believe that GAT is a promising candidate for mass-trapping intervention in urban settings, but a source reduction intervention should be made prior trap deployment. Therefore, we suggest future field studies to confirm the use of GAT as a complementary tool in vector control activities.

## Introduction

About half of the world’s population lives in areas where the risk of infection by dengue, Zika, yellow fever or chikungunya viruses is extremely high [1, 2]. It is estimated that dengue causes about 100 million symptomatic infections and 10,000 deaths annually in over 125 countries [3]. These arboviruses are important viral mosquito-borne diseases, which are mainly transmitted by *Aedes aegypti* mosquitoes, and except for yellow fever (for which there is a vaccine), the prevention and reduction of transmission are dependent on mosquito control measures.

Existing *Ae*. *aegypti* control methods that focus on removal or treatment of breeding sites and application of adulticides [4] often have unsatisfactory results, facing several challenges that can compromise its effectiveness. Gravid females can move from an area with low density of breeding sites to a neighboring areas with a higher availability of breeding habitats, therefore increasing their dispersal [5, 6]. Additionally, females can also lay their eggs in the most unlikely sites in areas often inaccessible by humans [7, 8]. The use of adulticides also presents limitations, including the rapid development of resistance to insecticides [9] and operational difficulties, in addition to being recommended only in response to outbreaks [4].

New approaches targeting *Ae*. *aegypti* adults are currently being evaluated to either reduce or replace wild mosquito populations. The strategies of releasing genetically modified mosquitoes, sterile male release or releasing mosquitos infected with *Wolbachia* bacteria could either suppress wild populations or decrease their ability to transmit arboviruses [10, 11]. Mass-trapping intervention is also an important alternative strategy for mosquito population reduction and subsequently reduces virus transmission [12–15].

Several traps have been tested as control tools, most of which function based on “lure and kill”, attracting and then killing gravid *Ae*. *aegypti*, thus reducing adult mosquito populations and their offspring [13, 16–18]. These traps have the advantage of capturing gravid females that have taken at least one blood-meal and are more likely to be infected with virus than parous unfed females. Another advantage of using this strategy is that trapped mosquitoes can be analyzed to detect arbovirus infection, thus indicating the level of viral circulation within the collection area [12, 19, 20].

The Gravid *Aedes* Trap (GAT) [21] is a passive trap that has been shown in several studies to be effective for the capture of gravid *Ae*. *aegypti* mosquitoes [21–26]. The GAT has outperformed different types of sticky ovitraps, collecting significantly more *Ae*. *aegypti* females than the MosquiTRAP and the Double Sticky Ovitrap in a field study in Australia [20]. The GAT also outperformed an Autocidal Gravid Ovitrap (AGO) in northeastern Florida [27]. When the GAT was compared with the BG-Sentinel trap (BGS), it caught overall fewer *Ae*. *aegypti* [20, 28, 29] and *Aedes albopictus* [30] mosquitoes than the BGS. However, the GAT caught significantly more gravid females than the BGS.

The GAT was evaluated as a novel tool for *Ae*. *albopictus* mosquito control by a small community in the USA guided by scientific advisors, which provided strong evidence that the GAT trap can significantly reduce *Ae*. *albopictus* biting pressure over time by mass trapping [24]. The GAT is a simple, lightweight, low-cost trap that doesn’t require electricity, with potential as an alternative control method. However, currently there is no evidence that *Ae*. *aegypti* populations can be suppressed by using the GAT as a tool for mass trapping and this investigation is an initial assessment of a series of studies to evaluate the feasibility of the GAT for this purpose. We evaluated, therefore, the performance of the GAT to control gravid *Ae*. *aegypti* in semi-field conditions using a simulated outdoor environment.

## Methods

### Study area

The study was conducted at the semi-field system (SFS) facility located at Biological Sciences Institute, Federal University of Minas Gerais (UFMG) in Belo Horizonte, southeastern Brazil. The SFS is located outdoor in a ventilated, wooded and shaded area, and consists of a large screened-chamber, with walls made of UV-resistant shade netting, measuring 14m x 7m [31]. During the study period the mean temperature was 26.4 ± 1.16°C and the relative humidity was 71.5 ± 4.84%. Inside the main SFS cage, two smaller chambers (6m x 3m x 3m) were used to conduct the experiments, in which two identical settings were arranged to simulate an outdoor environment. The simulated outdoor environments (SOE) were prepared with plastic tables and chairs, wooden chairs, ornamental plants (such as *Dypsis lutescens*, *Dracena Sanderiana*, *Chamaedorea elegans*, *Rhapis excels*, etc), metal shelves, bricks and wooden boards. Some furniture was made with cardboard boxes and covered with craft paper in order to obtain identical visual stimulation in both cages.

### Mosquitoes

*Aedes aegypti* mosquitoes (F3-F4) were reared under standard insectary conditions (27±2°C, 80±10% RH and 12:12h photoperiod) and fed with aquatic reptile food (Reptolife^®^, Alcon, Brazil). In a separate room, adult mosquitoes were maintained at an average temperature of 27°C, relative humidity of 70–90%, and a photoperiod of 12:12 (L:D). Adult male and female mosquitoes were kept in 30 x 30 x 30 cm screened polypropylene cages (Bugdorm-1^®^, Mega View Science Education Services, Taiwan) with a 10% sucrose solution soaked on a cotton wick as a food source. Female mosquitoes aged 5–7 d old were fed with chicken blood (*Gallus domesticus*—obtained from a chicken slaughterhouse) using a membrane feeder system [31, 32]. Cohorts of one hundred gravid *Ae*. *aegypti* females were then released in the SOE 3-days after the blood meal for each of the experiments here.

### Artificial breeding sites

Four types of containers were installed as artificial breeding sites in each simulated outdoor environment (SOE) chamber, all with a mat black color, differing only in dimension, denominated as: (a) small (SB– 6.5 x 9 cm); (b) regular size “ovitrap” (OV– 9 x 12.5 cm); (c) medium (MB– 20.5 x 20 cm); and (d) large (LB– 28 x 26 cm). Each breeding container had 40% of its total volume filled with tap water (80 mL, 400 mL, 2000 mL, and 5600 mL for the SB, OV, MB, and LB, respectively). Moreover, wooden paddles were added as oviposition stimulants to each breeding site and their sizes were calculated according to the dimension of the containers, as follows: SB and OV (3 x 10 cm), MB (7 x 16 cm), and LB (9 x 21 cm).

### Traps

#### Gravid *Aedes* trap (GAT)

GAT (BG-GAT, BioGents HmGb, Regensburg, Germany) is a “passive” mosquito trap that kills mosquitoes resting on an insecticide-treated plastic mesh screen positioned inside the translucent housing of the trap. The base consists of a black matte 20cm height x 30cm top diam x 24cm bottom diam. [21]. The trap is black with a translucent upper chamber, and a black nylon screen separating them. For this study, the GAT was used with 2.5g of alfalfa infusion (*Medicago sativa*) pellets and 3L of water (aged for 7 days). In addition, the surfaces of the translucent chamber and the nylon screen were sprayed with a commercial insecticide (SBP^®^ Reckitt Benckiser Group Plc: 0.02% imiprothrin; 0.05% permethrin; 0.1% biotrin), following the manufacturer’s recommendation every four weeks [20, 33].

#### BG-Sentinel trap (BGS)

BG-Sentinel-1 trap (BGS) (BioGents HmGb, Regensburg, Germany) is a collapsible trap, with a cylindrical shape measuring 35cm in diameter and 40cm in height [34]. The trap has a 12-volt electric fan and thus, it is an active trap, which produces a downwards airflow for sucking approaching mosquitoes into a collection bag. No synthetic human attractants were used in these traps.

#### BG-Mosquitito trap

BG-Mosquitito (BioGents HmGb, Regensburg, Germany) uses the BGS airflow concept, but in a more compact and lightweight design (0.25 kg) with a conical shape [35]. The trap body consists of a blue fabric cone and the top is a reflective white fabric with 35cm diameter. Unlike BGS, BG-Mosquitito is designed to be suspended 1 m above the ground and mosquitoes captured must first pass through the fan before being sucked into a collection bag inside the trap.

### Experimental setup

**Fig 1:**
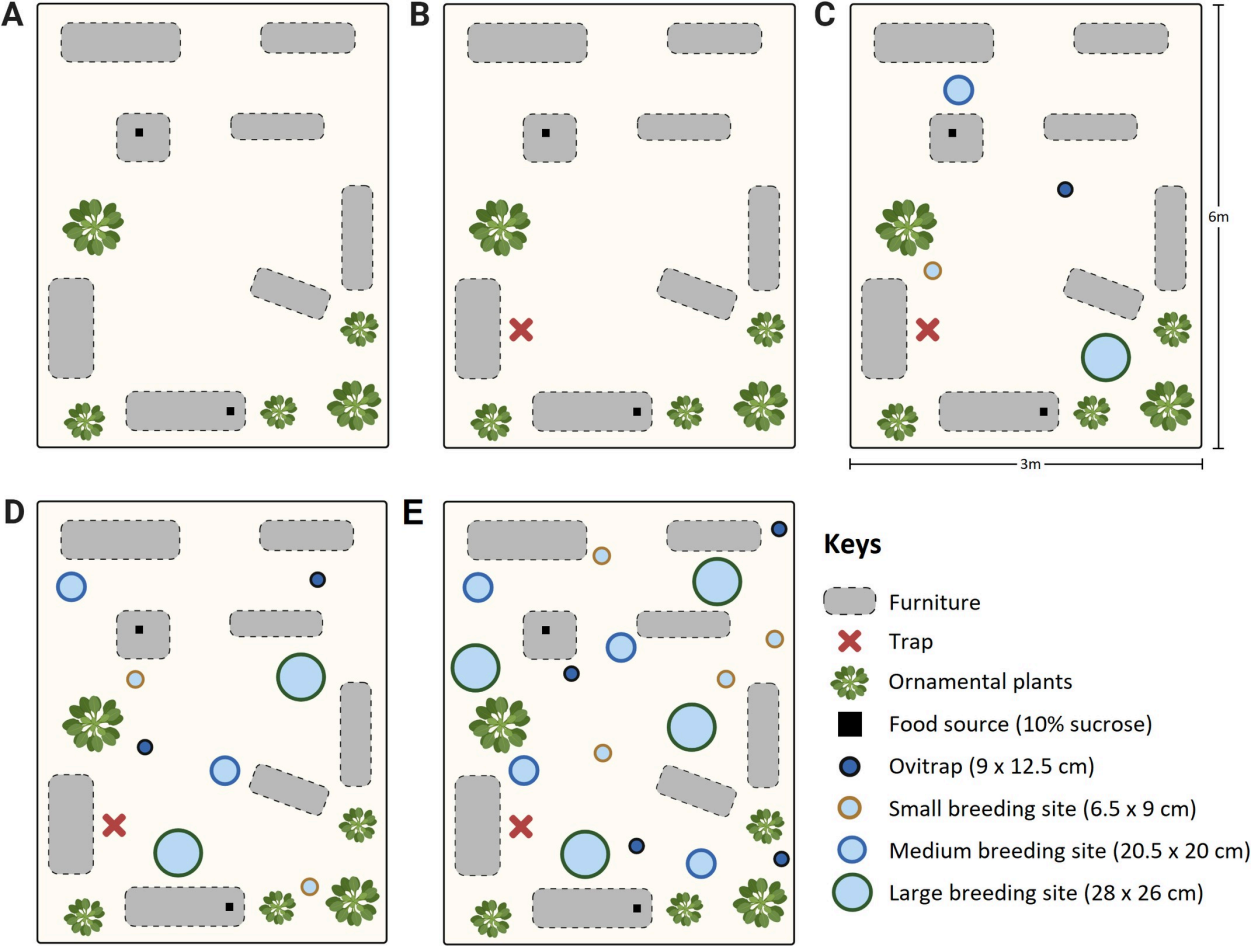
Illustration of the experimental setup showing the distribution of breeding sites and trap position in the simulated outdoor environment. Settings (A) without trap, with (B) 0, (C) 4, (D) 8 or (E) 16 containers of different sizes. Figure created using BioRender (https://biorender.com).

To assess the GAT as a control tool for *Ae*. *aegypti* adult females, the ability of a single trap to recapture the mosquitoes was evaluated in a SOE. The impact of oviposition sites on the capture rates was also evaluated using four arrangements of breeding site with different numbers (0, 4, 8 and 16) and sizes in two SOEs. The breeding sites were homogeneously distributed and the GAT was placed in identical position in each SOE (Fig 1). Each set of breeding site densities were considered as a treatment, which were separately evaluated (except for tests with no breeding sites) simultaneously in both SOEs. A single BGS trap was also tested separately in the SOE as a “gold standard” for all treatments.

#### Evaluation of Gravid *Aedes* Traps for sampling *Aedes aegypti* in environments with different breeding site densities

Both traps were separately evaluated according to the arrangements showed in Fig 1. For each treatment both SOEs were used concomitantly. However, while the traps (BGS or GAT) were deployed in only one SOE to recapture the mosquitoes, the other SOE was maintained without any of the evaluated traps (control treatment). In each SOE, approximately 100 gravid *Ae*. *aegypti* females were released and the experiments performed over 48 hours, starting and ending between the 3rd and 6th hour of the photophase (07:00h A.M.– 01:00h P.M.), respectively. Therefore, the experiments ended 4–5 days after the mosquitoes had received a blood meal. Two containers (200 mL) with cotton soaked in 10% sucrose solution were placed inside the SOEs as a food source for the mosquitoes during the experiment. At the end of the experiment, the traps were emptied, and all mosquitoes recaptured in each trap were counted and transported to the laboratory for parity studies by dissection of the ovaries [36]. Female mosquitoes that had more than five eggs retained in their ovaries were considered "gravid", whereas those with less than five eggs in their ovaries were considered parous [37].

Four replicates were completed for each treatment and the traps were installed alternately in each SOE to annul any position-related biases. At the end of every replicate experiment, all mosquitoes were removed from the SOEs using three BG-Mosquitito traps in each SOE for 24 h. In addition, before starting each experiment, the SOEs were thoroughly inspected and any remaining mosquitoes were removed manually with aspirators.

#### Evaluation of *Aedes aegypti* breeding productivity

At the end of the experiments, breeding sites and wooden paddles with eggs were collected to evaluate the productivity of each breeding site. The eggs in the water and attached to the wooden paddles were quantified using a stereo light microscope (x 20) and a manual counter. After counting the eggs, the water from each breeding site was transferred to a similar sized container for observing larval development until adult stage, which was characterized a productive breeding site (PBS). Each container was covered with a white fabric screen secured onto the container with an elastic band to prevent oviposition by other mosquitoes. The wooden paddles were maintained in the laboratory for three days to enable the eggs to complete embryonic development. Subsequently, the paddles were returned to their respective breeding site, which were then were maintained under semi-field conditions for 24 days (21 days for the paddles) to determine the productivity of each breeding site from egg hatching, larval and pupal counts. In order to study the natural larval development in the breeding sites, no nutrient was provided. Therefore, the nutrient source in all of the breeding sites was probably provided from organic matter released by the wooden paddle. After this period, all the larvae from first to fourth instars, pupae and pupal exuviae were counted. The pupal exuviae were used to evaluate the number of adults produced at by each PBS.

### Data analysis

To evaluate the effectiveness of the traps in collecting mosquitoes, the response variables were: (a) number of captured females/number of released females; (b) number of captured females for each parity/reproductive state/total number of captured females; (c) number of eggs laid in each breeding site/total number of eggs laid; (d) number of live individuals in the PBS/total number of eggs laid in the experimental breeding sites. For these last response variables (c and d), the explanatory variable was the dimension of the breeding site. The data were analyzed using GLM with quasi-binomial error structure. The models were compared by ANOVA contrast analysis followed by a Chi square test, both with 95% Confidence Intervals (CI).

Other response variables considered in the analysis were (a) number of females released in each replicate; and (b) total number of eggs laid in each breeding site in the presence or absence of a trap. For these response variables, the explanatory variable was the treatment evaluated (number of breeding sites available in the SOE). The data were analyzed using GLMs with Gaussian distribution. The models were compared by ANOVA contrast analysis followed by F-test, both with 95% Confidence Intervals (CI).

The analysis was performed using R software version 3.3.2 and the graphics were produced using GraphPad Prism software version 5.0 (GraphPad Software, San Diego California USA) and BioRender.com (https://biorender.com).

## Results

### *Aedes aegypti* recapture by the Gravid *Aedes* Trap and BG-Sentinel in environments with different breeding site densities

**Fig 2:**
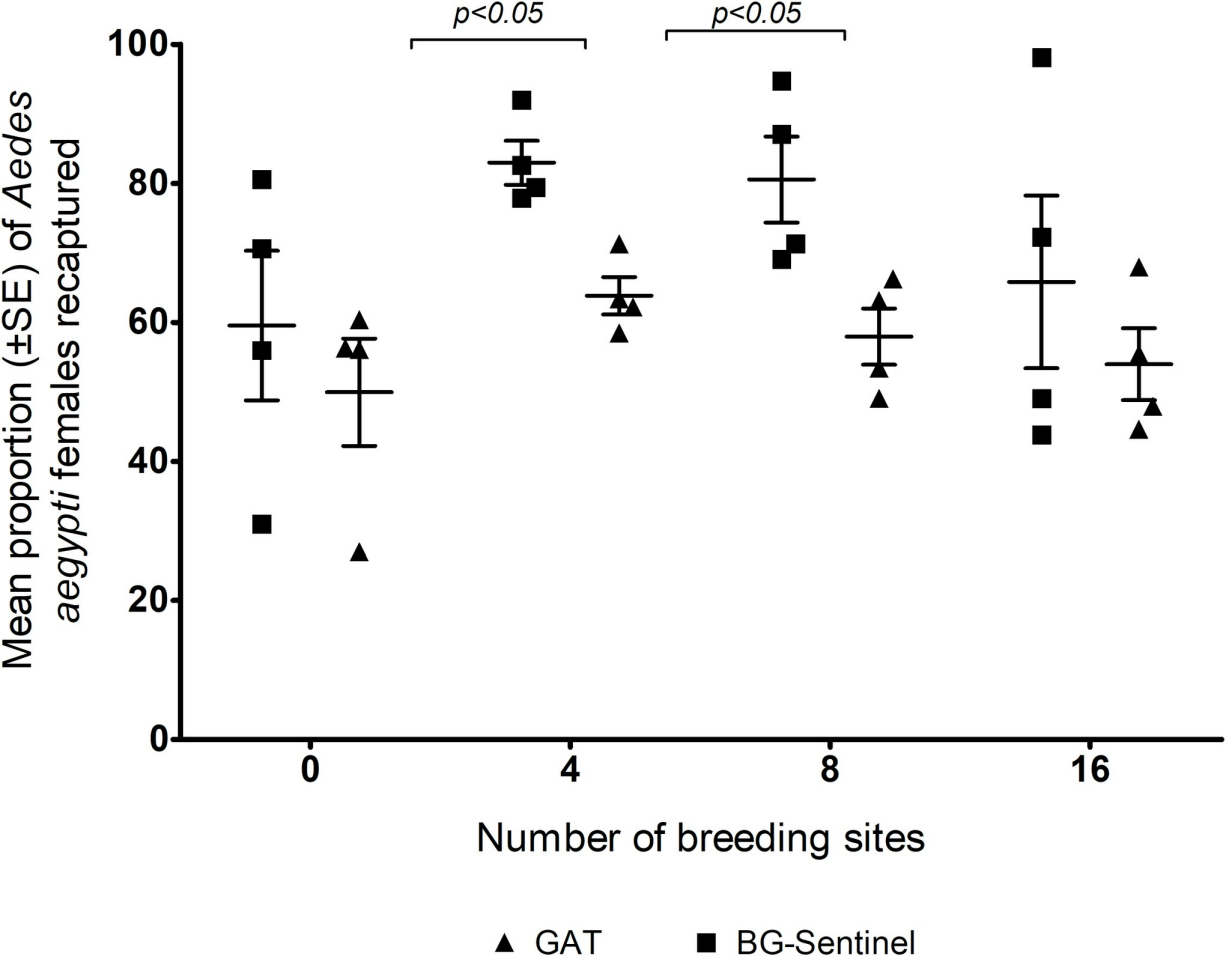
Mean percentage (± SE) of *Aedes aegypti* females recaptured by the BG-Sentinel trap (A) or by the Gravid *Aedes* Trap (B) and the comparison between both traps (C) in a simulated outdoor environment with 0, 4, 8 or 16 breeding sites per environment. Horizontal lines on Panel C show the significant difference for the GAT and the BGS in experiments with 4 and 8 breeding sites per environment.

Throughout the study period, approximately 6,400 *Ae*. *aegypti* females were released into the semi-field chambers. In experiments when no breading container was available, the BGS trap recaptured from 30 to 82%, whereas when there were 16 breeding sites, the recapture rate ranged from 43% to 100%. There was a narrow variation for 4 and 8 breeding sites. The mean recapture rates of the BGS trap ranged from 60 to 82% depending on the number of breeding sites per SOE (Fig 2A). The highest mosquito catches using the BGS were obtained in the SOE containing 4 breeding sites (82.9 ± 3.16%), followed by the experiment with 8 breeding sites (80.6 ± 6.20%). However, these results were not significantly different from one another (DF = 2.787; X^2^ < 0.001; p = 0.949).

The recapture rate of the GAT changed slightly regarding the viability of breeding container and the results were consistent throughout the number of breading containers. The minimum recaptured rate was observed for the experiment when no containers were available (20%) and the maximum recapture rate (71%) was seen with 4 breading containers per SOE. The overall mean recapture rate ranged from 50% - 65% and there was no significant difference (DF = 0.717; X^2^ < 0.001; p = 0.968) between experiments with different numbers of breeding sites in the SOE (Fig 2B*)*. Although the BGS recaptured higher numbers of mosquitoes than the GAT in all the SOEs evaluated, when comparing both traps a significant differences was only observed in environments containing 4 breeding sites (DF = 0.502; X^2^ = -0.393; p < 0.001) and 8 breeding sites (DF = 0.892; X^2^ = -0.488; p < 0.005), all other treatments were not significantly different (Fig 2C).

### Reproductive status of *Aedes aegypti* recaptured by the Gravid *Aedes* Trap, BG-Sentinel and BG-Mosquito trap

**Fig 3:**
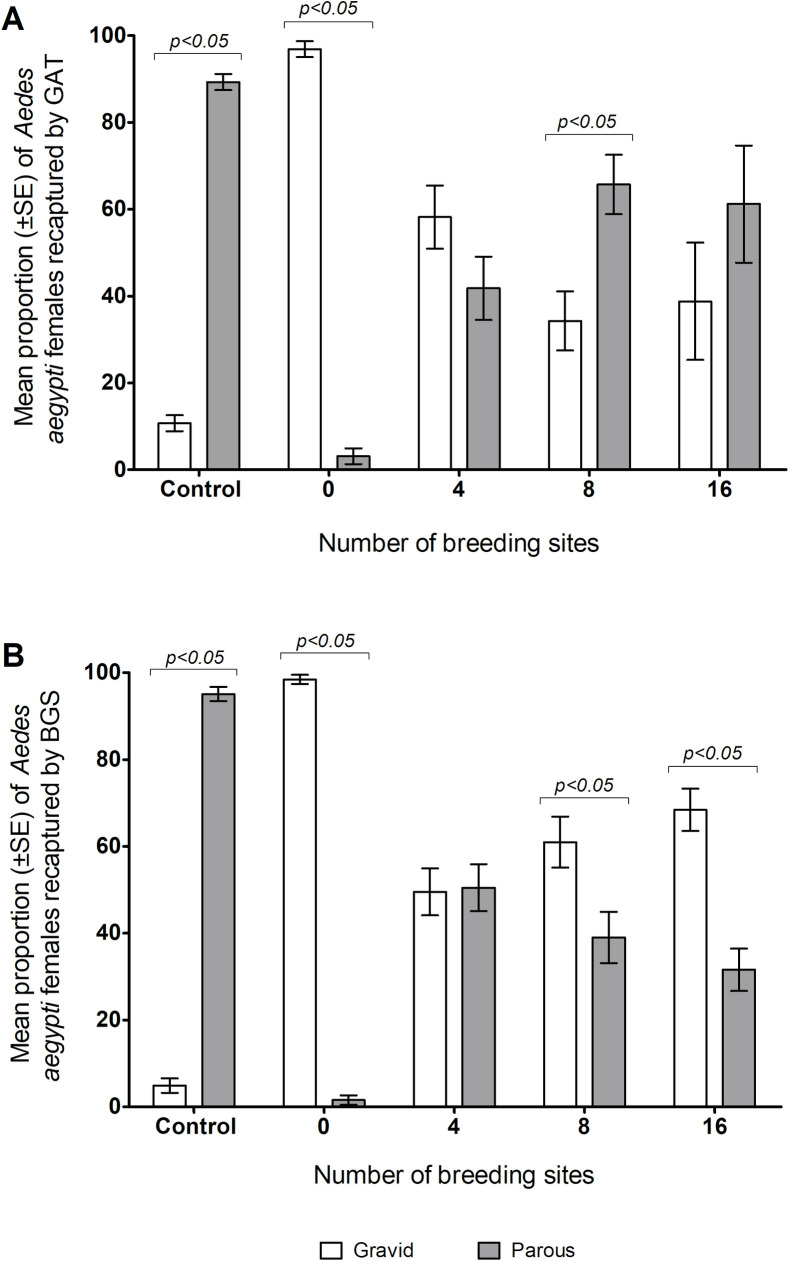
Reproductive status of *Aedes aegypti* females recaptured by the Gravid *Aedes* Trap (A) and the BG-Sentinel (B) in a simulated outdoor environment with 0, 4, 8 or 16 breeding sites per environment. In the control treatments (no GAT or BGS present), the females were recaptured using BG-Mosquitito traps. Horizontal lines on top of the bars show the significant difference for gravid and parous females recaptured in the control environments, with 0, 8 breeding sites and 16 breeding sites.

As expected, in the control treatment the BG-Mosquitito traps recaptured significantly more parous mosquitoes than gravid mosquitoes after 48 h and, the GAT or BGS recaptured significantly more gravid mosquitoes (>95%) than parous ones when no breading sites were offered (Fig 3). It was observed that the BGS trap had a reduced recapture rate of gravid mosquitoes as the number of breading sites increased, whereas the GAT had increased recapture rates when using from 4 to 16 breading sites.

### Oviposition behavior of *Aedes aegypti* in environments with different breeding site densities

The number of eggs laid by *Ae*. *aegypti* females in each treatment, with or without the presence of traps, is shown in Table 1. Interesting, the presence of the BGS reduced the rate of egg laying by 44.3% to 56.9% in SOEs with increasing numbers of breading sites. There were significant differences between the SOEs with and without traps when using 8 breading sites (DF = 2898102; F = 6.353; p = 0.045) and 16 breading sites (DF = 9862292; F = 14.819; p < 0.05). However, no significant difference was observed with 4 breeding sites and its respective control (DF = 2747289; F = 2.564; p = 0.161).

**Table 1:** Mean number of eggs laid by *Aedes aegypti* females and the relation to the presence of a BG-Sentinel trap (BGS) or a Gravid *Aedes* trap (GAT), in a simulated outdoor environment with 4, 8 or 16 breeding sites.

Treatment		Mean number (±SE) of females released	Number of eggs laid (mean ± SE)	Reduction of eggs laid (%)
Number of breeding sites	Traps			
4	No trap	98.5 ± 2.72	5,786	44.3
			(1,446.5 ± 341.1)^a^	
	BGS	97.3 ± 2.29	3,221	
			(805.3 ± 209.9)^a^	
8	No trap	98.5 ± 1.19	7,376	46.8
			(1,844.0 ± 206.3)^a^	
	BGS	96.5 ± 1.71	3,923	
			(980.8 ± 273.4)^b^	
16	No trap	100.0 ± 6.04	13,160	56.9
			(3,290.0 ± 466.9)^a^	
	BGS	100.3 ± 5.79	5,666	
			(1,416.5 ± 137.5)^b^	
4	No trap	100.8 ± 1.88	12,715	11.5
			(3,178.8 ± 133.6)^a^	
	GAT	100.0 ± 4.45	12,252	
			(2,813.0 ± 1181.9)^a^	
8	No trap	98.8 ± 2.50	10,798	11.9
			(2,699.5 ± 424.5)^a^	
	GAT	101.5 ± 1.66	9,514	
			(2,378.5 ± 255.5)^a^	
16	No trap	100.0 ± 4.81	9,265	0.7
			(2,316.3 ± 493.7)^a^	
	GAT	98.5 ± 2.72	9,197	
			(2,299.3 ± 356.3)^a^	

Different letters indicate significant difference between each treatment group with and without traps (p <0.05).

Differently, the presence of the GAT only slightly reduced the mean proportion of eggs, which ranged from 0.7 to 11.9% and no significant difference was observed between any of the treatments and their respective controls [4 breading sites (DF = 15386589; F = 0.011; p = 0.922), 8 (DF = 3151698; F = 0.420; p = 0.541) and 16 (DF = 4449544; F < 0.001; p = 0.979)] (Table 1).

**Fig 4:**
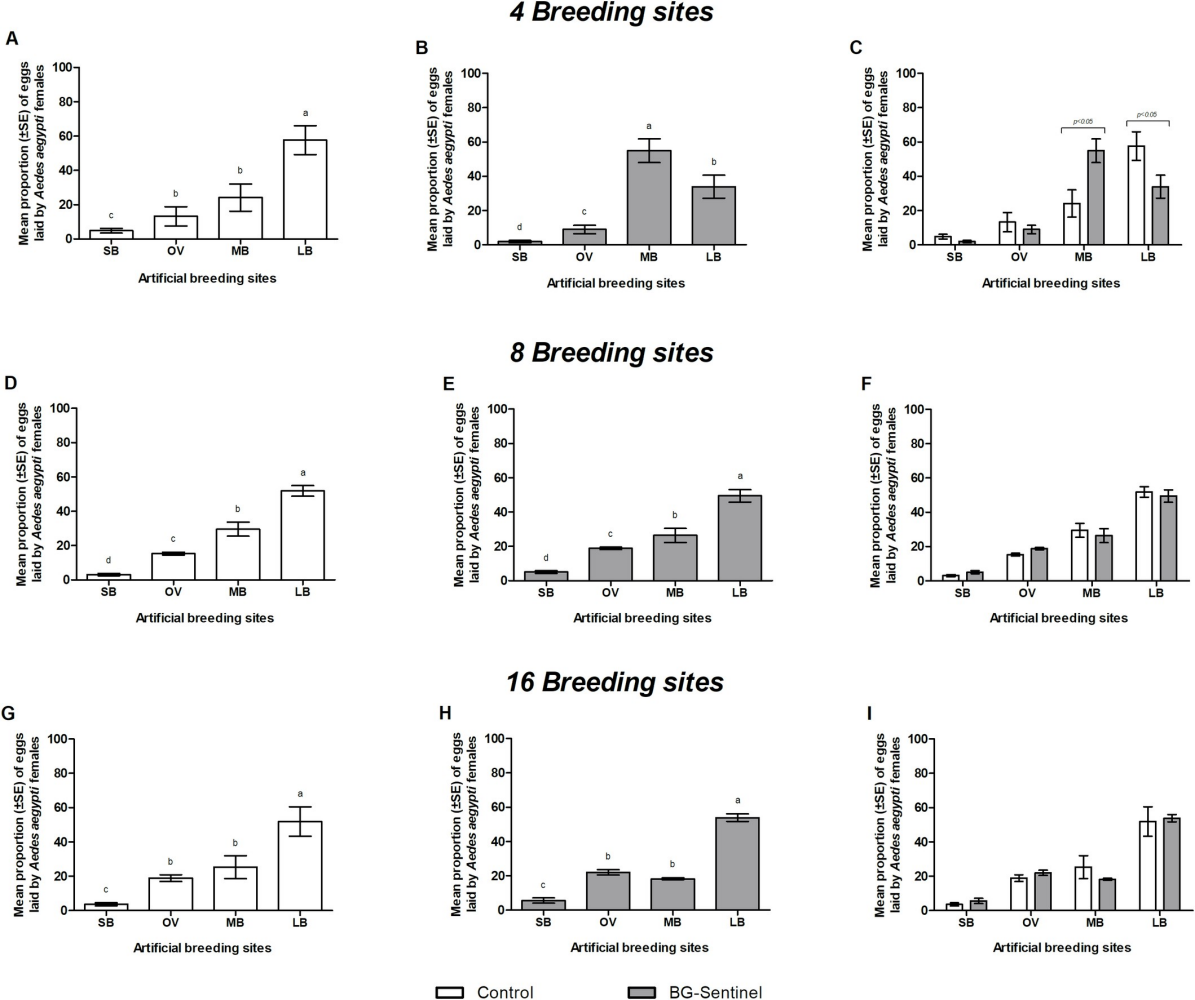
Mean percentage (± SE) of eggs laid by *Aedes aegypti* females in breeding sites with different sizes in simulated outdoor environments with 4, 8 or 16 breeding sites and the presence (B, E and H) or absence (A, D and G) of a BG-Sentinel trap (BGS). Panel C, F and I shows the comparison for percentages of eggs laid in different breeding sites with and without a trap. SB: small artificial breeding site (6.5 x 9 cm); OV: regular size ovitrap (9 x 12.5 cm); MB: medium artificial breeding site (20.5 x 20 cm); LB: large artificial breeding site (28 x 26 cm). Different letters on top of the bars for panels A-B, D-E and G-H indicate significant differences (p <0.05). Horizontal lines on top of the bars in Panel C show the significant difference for MB with and without the BGS in the environment and for LB with and without the BGS trap.

**Fig 5:**
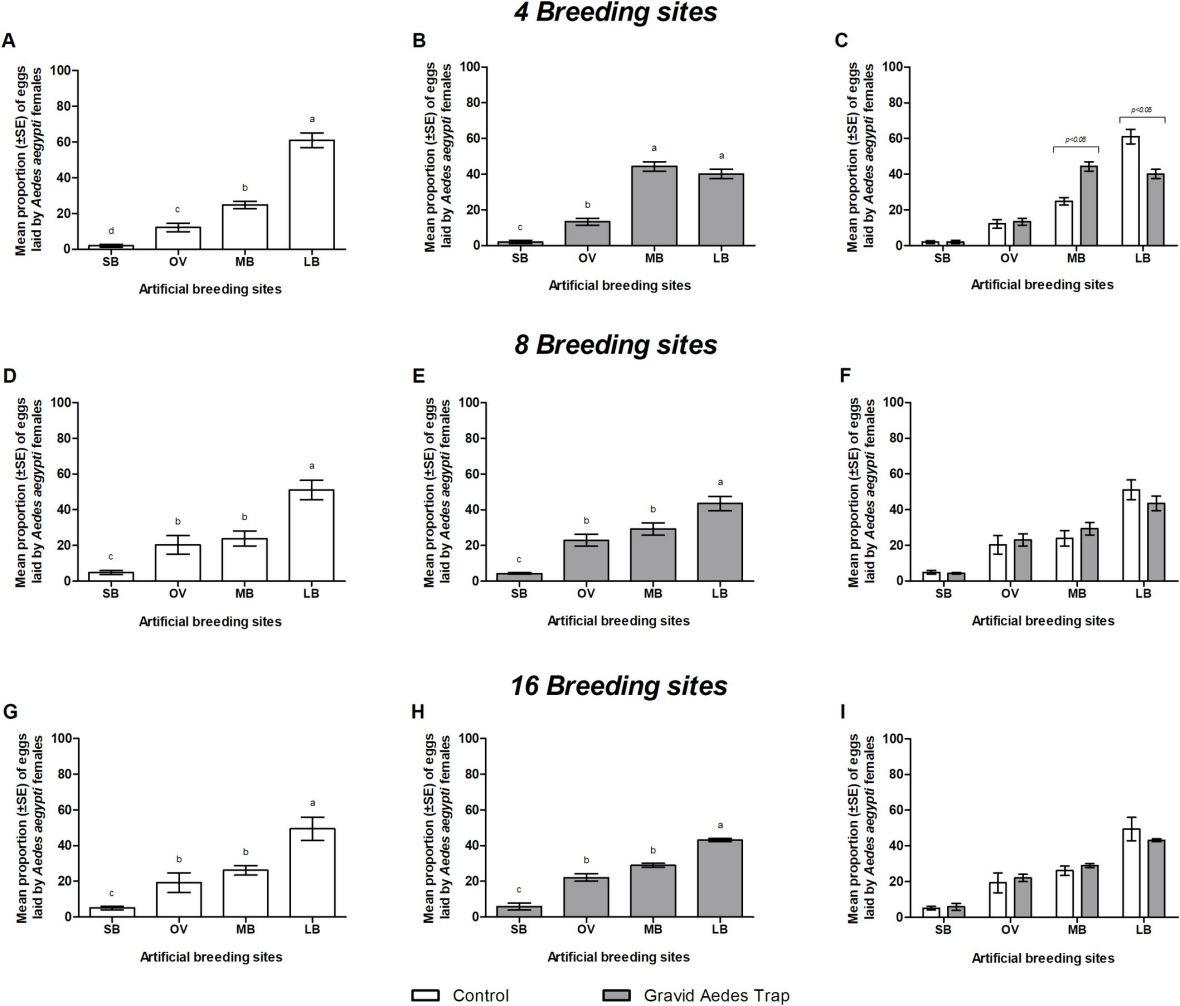
Mean percentage (± SE) of eggs laid by *Aedes aegypti* females in breeding sites with different dimensions in simulated outdoor environments with 4, 8 or 16 breeding sites and the presence (panels B, E and H) or absence (panels A, D and G) of the Gravid *Aedes* trap (GAT). Panels C, F and I shows the comparison among eggs laid in different breeding sites according to the environments with and without traps. SB: Small artificial breeding site (6.5 x 9 cm); OV: Regular size ovitrap (9 x 12.5 cm); MB: Medium artificial breeding site (20.5 x 20 cm); LB: Large artificial breeding site (28 x 26 cm). Different letters at the top of the columns in panels A-B, D-E and G-H indicate significant differences (p <0.05). Horizontal lines on top of the bars in Panel C show the significant difference for MB with and without the BGS in the environment and for LB with and without the BGS trap.

Interestingly, *Ae*. *aegypti* laid eggs in almost all breeding sites available in all the treatments (Figs 4 and 5). It was observed in the control treatment that the proportion of eggs laid increased significantly with increasing the size of breading site (Figs 4A, 4D, 4G, 5A, 5D and 5G). However, the oviposition occurred heterogeneously within the environments when a BGS (Fig 4) or a GAT was present (Fig 5).

In tests to evaluate the effect of the BGS in reducing the number of eggs laid by *Ae*. *aegypti*, the results followed the same trend as that of the control treatment, in which the proportion of eggs laid increased significantly with increasing the size of breading site (Fig 4E and 4H), except for 4 breading sites, in which MB received the highest percentage of eggs (55 ± 6.9%) (DF = 1.334; X^2^ = - 1.106; p < 0.001) (Fig 4B). In the environment with 4 containers without the BGS, however, there was no significant difference between the number of eggs laid in OV and MB (p > 0.05) (Fig 4A). Likewise, no significant differences were found between eggs laid in OV and MB in scenarios with 16 breeding sites, both with and without the BGS (p > 0.05) (Fig 4G and 4H).

When the effect of the presence or absence of BGS in the reduction of the percentages of eggs laid in each group of breeding sites was compared (Fig 4C, 4F and 4I). Significant differences were observed only in the simulation with 4 MBs (DF = 3.181; X^2^ = 1.231; p < 0.001) and LBs (DF = 3.181; X^2^ = 1.231; p < 0.001) (Fig 4C).

Similar results were observed in experiments with the GAT, where large size breeding sites (LB) also received the highest percentage of eggs (p < 0.05) (Fig 5E and 5H), except for the environment with 4 breeding sites + GAT (Fig 5B). There was no significant difference between the number of eggs laid in LB and MB (p > 0.05). In experiments with 8 and 16 breeding sites, regardless the presence of the GAT, the percentage of eggs laid in the OVs did not differ significantly from the percentage of eggs laid in the MBs (p > 0.05) (Fig 5D, 5E, 5G and 5H, respectively*)*.

When the proportion of the eggs was compared with and without trap, the MBs and LBs were significantly influenced by the presence of the GAT in experiments with four breeding sites (p < 0.05) (Fig 5C). The MBs received more eggs in the environment with GAT than the environment with no trap (DF = 0.770; X^2^ = 0.365; p < 0.001). However, for LBs, fewer eggs were laid when the GAT was present (DF = 0.785; X^2^ = 0.380; p < 0.001). In the other experiments with 8 and 16 breeding sites, no significant differences were found in the presence of the GAT (p > 0.05) (Fig 5F and 5I).

### *Aedes aegypti* breeding productivity

**Fig 6:**
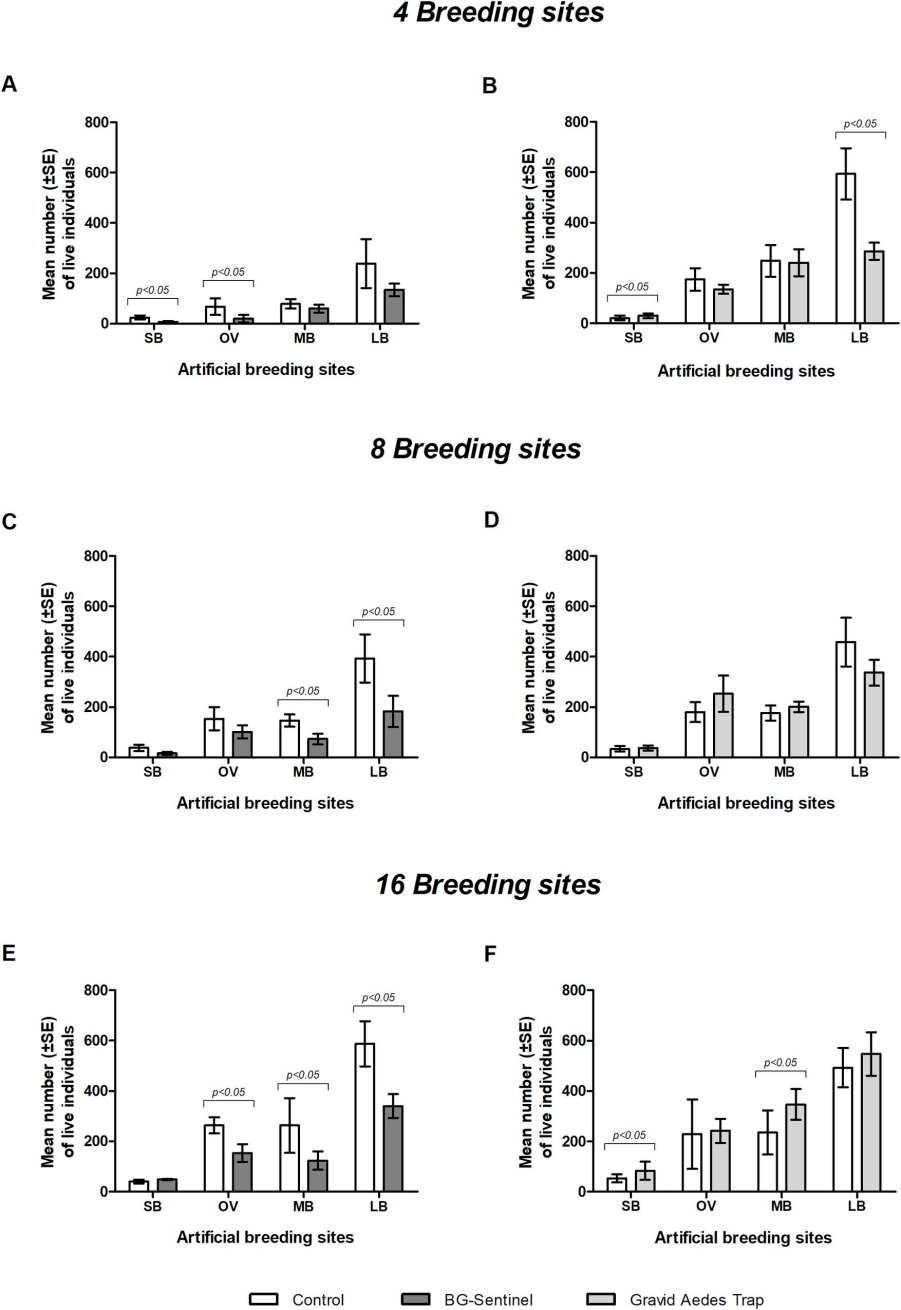
Mean percentage (± SE) of breeding productivity (live larvae, pupae and adults after 24 days) of containers with different dimensions in a simulated outdoor environment with 4, 8 or 16 breeding sites and the presence or absence of BG-Sentinel trap (panels A, C and E) or Gravid *Aedes* trap (panels B, D and F). SB: Small artificial breeding site (6.5 x 9 cm); OV: Regular size ovitrap (9 x 12.5 cm); MB: Medium artificial breeding site (20.5 x 20 cm); LB: Large artificial breeding site (28 x 26 cm). Horizontal lines on top of the bars in Panel B show the significant difference for MB with and without the GAT (control) in the environment.

*Aedes aegypti* breeding productivity in containers of different sizes in SOEs in the presence of BGS or GAT is shown in Fig 6. In general, the BGS trap reduced the productivity of *Ae*. *aegypti*, regardless of the breading sites number and sizes. Significant reduction of productivity compared with the control treatment were observed at SB and OV for 4 breading sites (Fig 6A), and 8 or 16 breeding sites for MB and LB containers in SOEs (p < 0.05) (Fig 6C and 6E).

However, the GAT has little effect on the reduction of the *Ae*. *aegypti* productivity. The only significant reduction in productivity compared with the control treatment (p < 0.05) were observed with 4 SB or LB breeding sites (Fig 6B). The productivity of containers of different dimensions did not differ significantly in experiments with 8 breeding sites (p = 0.514), whereas in SOEs with 16 breeding sites the productivity were significantly higher for the GAT (p < 0.05) (Fig 6D and 6F, respectively).

## Discussion

The development and use of traps to capture adult *Ae*. *aegypti* are included in the recommendations of the World Health Organization for the control of *Ae*. *aegypti*-transmitted viruses [38, 39]. Adult mosquito traps are generally used in vector surveillance to monitor the distribution, abundance and infection rates of vector populations. Several traps have been evaluated recently for vector control by mass-trapping, such as the active BGS trap [14], the passive AGO trap [26, 40] and the passive GAT [13, 24]. Passive traps can control gravid *Aedes* mosquitoes that have a higher chance of being infected with arbovirus than those found in the general adult mosquito population, since they have had contact with vertebrate hosts during a bloodmeal. If gravid mosquitoes are regularly removed from the environment, there will be no new offspring and therefore a swift reduction in the population will occur [13]. Consequently, the traps could reduce disease transmission by lowering vector densities below the transmission threshold and, thus, shifting the age structure and reducing the abundance of infectious vectors. The passive gravid traps, AGO and GAT, are produced commercially and have been used on a large scale [17, 24, 26, 29, 40, 41]. They are simple, low-cost, specific for *Aedes* mosquitoes, and are strong candidates for mass-trapping interventions.

The use of semi-field systems is important to study mosquito behavior as several factors can be controlled such as temperature, humidity, number and age of mosquitoes released into the environments [42], as well as number and size of breading sites on offer. The current study used a semi-field system that simulated a Brazilian urban house and yard, which is the primary habitat of *Ae*. *aegypti*, with scenarios of furniture and plants and varied densities and sizes of breeding sites. Therefore, we could investigate the response of the mosquitoes to two different traps in relation to the different scenarios. Two identical outdoor environments were compared simultaneously, one with a trap and the other without a trap. The environments were used to compare mosquitoes recapture rates when deploying the GAT and the BGS, which was used for comparison. This approach was conducted in order to understand how a passive trap such as the GAT, can reduce the population of gravid *Ae*. *aegypti* in a controlled environment.

We used the BGS trap as a so called “gold standard” trap as several studies have shown that the BGS is more efficient for sampling, monitoring and controlling *Aedes* population in the field than other commercially available traps [14, 15, 17, 34]. In our study, we established the recapture rates of blood fed *Ae*. *aegypti* over 48 hours to make sure that the gravid mosquitoes would oviposit their eggs using the “skipping behavior” [43]. Passive traps are usually inspected on a weekly basis, whilst in field situations, the BGS is usually run for 24h, but here we used a 48 h period for direct comparison between the efficacy of these traps. Although these traps use fundamentally different concepts to catch mosquitoes, both traps use olfactory (in this study we only used an olfactory stimulus for the GAT trap) and visual stimuli to lure mosquitoes. However, the BGS trap uses suction produced by an electrical fan to catch mosquitoes that approach the vicinity of the trap orifice, whereas the GAT relies only on mosquito oviposition site seeking behavior, to passively enter the trap, which is highly attractive for oviposition.

The present study showed that when gravid mosquitoes were released in the semi-field environment with no breeding sites available, the BGS trap recaptured higher proportion of mosquitoes than GAT, however, the results were not statistically different. The higher mosquito catches by BGS were expected as similar results have been previously described in other studies when the BGS outperformed the GAT in capturing *Ae*. *aegypti* in up to 50% in Queensland (Australia) [20] and approximately 5-fold in Florida (USA) [28]. Likewise, the superiority of BGS over GAT has also been reported for *Culex quinquefasciatus* in Australia, with the former capturing approximately 9-fold more mosquitoes [29]. On the other hand, firld study in Florida [28] also showed that when considering only the gravid *Ae*. *aegypti*, the mean numbers of mosquitoes collected by BGS and GAT were similar. Furthermore, in the study in Queensland [20] GAT collected 30% more gravid *Ae*. *aegypti* than the BGS [20].

In order to compare the efficacy of the GAT to recapture *Ae*. *aegypti* in different scenarios, different numbers and sizes of breeding sites were offered for oviposition. It was observed that this passive trap had an increased recaptured rate when 4 or 8 breeding containers were available. Interesting that the overall recapture rates by the BGS were higher than those observed using GAT, possibly due the suction created by the BGS electrical fan increasing capture rates of in mosquitoes flying close to the entrance of the trap.

In field studies, BGS traps were more specific for host seeking *Ae*. *aegypti* [44], parous and gravid mosquitoes [44, 45], whereas the GAT has been shown to attract only gravid *Aedes* mosquitoes [20]. In this study, when considering the parity status of the recaptured mosquitoes, as all released mosquitoes received a blood meal 3 days before the experiments, ovarian development was expected to be similar for all females. Therefore, we assumed that all recaptured female mosquitoes would be gravid or parous in their parity status. Over 95% of the mosquitoes recaptured by the BGS and the GAT were gravid when no breading containers were deployed. This suggests that the recaptured mosquitoes were not able to lay eggs because there were no breeding sites available. However, when breeding containers were available, competition between both traps and the containers was observed. The GAT showed efficiency to recapture more gravid mosquito when 4 containers were available and a decreased efficiency when the number of breeding container increased from 8 to 16. These results suggest that the GAT is most efficient for the capture of gravid *Ae*. *aegypti* only when no containers with water were available in the environment, because the water-filled containers would possibly compete with this gravid trap.

In this study the number of eggs laid during 48 h was also evaluated by sampling from different sized breeding containers (ovitraps). The presence of high numbers of eggs in the ovitraps indicated that the mosquitoes lay eggs before being caught in the traps. The presence of the BGS reduced the number of eggs in the SOEs for all treatments with different number of breeding sites. However, a significantly reduction was only observed when SOEs had 8 and 16 containers. These results suggest that the BGS recaptured female mosquitoes before laying all their eggs, with a reduction in the total number of eggs in SOEs with traps present when compared to the controls (no traps in the SOEs). This may reflect the effectiveness of the BGS electric fan, which is able to suck in mosquitoes approaching the traps and, thus, it is likely that females were recaptured whilst in flight, actively seeking breeding sites for oviposition.

The BGS contributed directly to a reduction of the mosquito population (reducing the number of eggs in breeding sites) and indirectly (reducing the number of adult females destined to lay eggs after the blood meal). The presence of the GAT, however, did not reduce the number of eggs laid during any of the experiments with different numbers of breeding sites when compared with the control treatment. These results suggested that gravid *Ae*. *aegypti* laid most of their eggs in the containers (breeding sites) in the SOEs before being recaptured by the GAT.

As gravid *Ae*. *aegypti* use “skip oviposition” behavior [43, 46–50], it is likely that they laid most of their eggs in the containers before visiting the GAT. Semi-field studies on the skip behavior of gravid *Ae*. *aegypti* has shown that a single female mosquito can oviposit in up to 8 breading sites, although most of the eggs (>50 eggs) are laid in a single breeding site [43]. In our study, a higher number of gravid mosquitoes were recaptured by the GAT + 4 breeding sites when compared to 8 and 16 breeding sites. It is possible that these mosquitoes were caught in higher proportions during the oviposition skip, as there were fewer breeding sites available. Although, the GAT was baited with hay infusion in this study, which makes it more attractive than the breeding sites with only water [51, 52], the insecticide impregnated inside of the translucent chamber of the GAT may have had a repellent effect at short-range and the flying mosquitoes skipped away from the trap to the other breeding sites available. Further studies will be conducted to investigate the attractivity of the GAT impregnated with insecticide.

Almost all the containers offered to the mosquitoes were used by the females to lay their eggs, the exception was in the experiments with 16 containers, where in two replicates, one with a GAT and the other with a BGS, in which only one breeding site had no eggs.

The results here show that the size of the breeding site is important. There is a tendency for larger containers to receive more eggs than smaller ones. The preference for larger containers displayed by female *Ae*. *aegypti* was also demonstrated under semi-field conditions [53] and in field studies [54]. This was probably because larger breeding sites are visually more attractive and more resistant to desiccation, in addition to have more resources available for the development of immature stages, such as space and organic matter [49, 54]. The greater number of eggs laid in larger containers and, consequently increase in breeding productivity is also shown in our results. If we project these findings to natural conditions it is likely that more adult individuals would be produced, and these breeding sites must be actively observed in mosquito surveillance and control programs, as no quantitative status is provided by House and Breteau index used to detect *Aedes*.

In conclusion, this study showed that the GAT was efficient in reducing gravid and parous *Ae*. *aegypti* populations in semi-field environments mainly when no breading sites are available, which leads us to suggest the evaluation to control wild populations of *Ae*. *aegypti* in urban settings, after source reduction. The GAT has many of the requirements for sustainable vector control i.e., simple, low cost, easy to set up and transport, electricity-free and targets adult *Aedes* females, as well as having the possibility of detection of mosquitoes positive for arboviruses [19]. The GAT costs about 5 times less that the BGS trap (available in https://biogents.com), therefore, it would be possible to deploy more GATs per area at much lower cost. However, it will be necessary to test the efficacy of higher numbers of GATs for this type of intervention. The GAT is an ideal candidate for mass-trapping interventions, although breeding site source reduction activities in the intervention areas are recommended before implementation of the GAT for controlling *Ae*. *aegypti*. These findings have a particular relevance given that, independent of the lower costs, the GAT is more acceptable by householders or the community because it does not require electricity [14, 26] and for mass-trapping interventions, the GAT does not required as much maintenance as the BGS. Further studies are now required to investigate the potential use of the GAT for the reduction *Ae*. *aegypti* populations in urban environments, which will be planned using the results of this study.

## Supporting information

S1 Table Summary of breeding productivity of containers with different dimensions in a simulated outdoor environment with 4, 8 or 16 breeding sites and the presence or absence of BG-Sentinel trap or Gravid Aedes trap. (DOCX)
