# Supplementary material for: Semi-field assessment of the Gravid Aedes Trap (GAT) with the aim of controlling Aedes (Stegomyia) aegypti populations
Source: PLoS One. 2021 Apr 29;16(4):e0250893. doi: 10.1371/journal.pone.0250893 (PMC8084243; doi:10.1371/journal.pone.0250893)
Supplement: S1 Table — (DOCX) [file pone.0250893.s001.docx]

**S1 Table. Summary of breeding productivity of containers with different dimensions in a simulated outdoor environment with 4, 8 or 16 breeding sites and the presence or absence of BG-Sentinel trap or Gravid Aedes trap.**

| **Number of breeding sites** | **Type of container** | **Treatment** | **Gravid Aedes Trap** | | | |  | **BG-Sentinel trap** | | | |
| --- | --- | --- | --- | --- | --- | --- | --- | --- | --- | --- | --- |
|  |  |  | **Number of eggs laid** | **Number of individuals* (mean ± SE)** | **Proportion of individuals (%)** | **P value** |  | **Number of eggs laid** | **Number of individuals* (mean ± SE)** | **Proportion of individuals (%)** | **P value** |
| 4 | SB | With trap | 209 | 121 (30.2 ± 9.7) | 57.89 | 0.010 |  | 77 | 30 (7.5 ± 2.9) | 38.96 | 0.005 |
|  |  | Control | 245 | 84 (21.0 ± 9.4) | 34.29 |  |  | 267 | 97 (24.2 ± 7.8) | 36.33 |  |
|  | OV | With trap | 1,451 | 543 (135.7 ± 17.8) | 37.42 | 0.370 |  | 320 | 82 (20.5 ± 14.5) | 25.63 | <0.001 |
|  |  | Control | 1,529 | 696 (174.0 ± 44.9) | 45.52 |  |  | 892 | 271 (67.7 ± 32.6) | 30.38 |  |
|  | MB | With trap | 5,566 | 964 (241.0 ± 54) | 17.32 | 0.525 |  | 1,819 | 240 (60.0 ± 15.9) | 13.19 | 0.394 |
|  |  | Control | 3,115 | 992 (248.0 ± 63.1) | 31.85 |  |  | 1,611 | 317 (79.2 ± 18.7) | 19.68 |  |
|  | LB | With trap | 5,026 | 1,145 (286.2 ± 34.1) | 22.78 | <0.001 |  | 1,005 | 537 (134.2 ± 24.9) | 53.43 | 0.273 |
|  |  | Control | 7,826 | 2,374 (593.5 ± 101.8) | 30.33 |  |  | 3,016 | 956 (239.0 ± 97.6) | 31.70 |  |
| 8 | SB | With trap | 413 | 150 (37.5 ± 10.4) | 36.32 | 0.819 |  | 180 | 65 (16.2 ± 6.1) | 36.11 | 0.072 |
|  |  | Control | 538 | 135 (33.7 ± 10.9) | 25.09 |  |  | 219 | 153 (38.2 ± 13.1) | 69.86 |  |
|  | OV | With trap | 2,266 | 1,012 (253.0 ± 71.7) | 44.66 | 0.268 |  | 744 | 406 (101.2 ± 25.4) | 54.57 | 0.210 |
|  |  | Control | 2 | 720 (180.0 ± 38.7) | 36.00 |  |  | 1,137 | 613 (153.2 ± 45.5) | 53.91 |  |
|  | MB | With trap | 2,791 | 806 (201.5 ± 21.1) | 28.88 | 0.169 |  | 1,022 | 295 (73.7 ± 21.2) | 28.86 | <0.001 |
|  |  | Control | 2,781 | 703 (175.7 ± 30.4) | 25.28 |  |  | 2,155 | 586 (146.5 ± 24.3) | 27.19 |  |
|  | LB | With trap | 4,044 | 1,347 (336.7 ± 51.7) | 33.31 | 0.176 |  | 1,977 | 731 (182.7 ± 62.3) | 36.98 | 0.038 |
|  |  | Control | 5,479 | 1,832 (458.0 ± 97.1) | 33.44 |  |  | 3,865 | 1,572 (393.0 ± 95.2) | 40.67 |  |
| 16 | SB | With trap | 555 | 335 (83.7 ± 36.2) | 60.36 | <0.001 |  | 304 | 193 (48.2 ± 2.6) | 63.49 | 0.158 |
|  |  | Control | 431 | 214 (53.5 ± 16.0) | 49.65 |  |  | 488 | 161 (40.2 ± 7.4) | 32.99 |  |
|  | OV | With trap | 1,990 | 968 (242.0 ± 47.9) | 48.64 | 0.213 |  | 1,239 | 615 (153.7 ± 35.3) | 49.64 | 0.023 |
|  |  | Control | 2,105 | 916 (229.0 ± 138.3) | 43.52 |  |  | 2,397 | 1,053 (263.2 ± 31.8) | 43.93 |  |
|  | MB | With trap | 2,678 | 1,386 (346.5 ± 61.1) | 51.76 | 0.035 |  | 1,046 | 493 (123.2 ± 36.3) | 47.13 | 0.014 |
|  |  | Control | 2,459 | 943 (235.7 ± 87.4) | 38.35 |  |  | 3,077 | 1,052 (263.0 ± 108.6) | 34.19 |  |
|  | LB | With trap | 3,974 | 2,189 (547.2 ± 86.7) | 55.08 | 0.617 |  | 3,077 | 1,358 (339.5 ± 47.4) | 44.13 | <0.001 |
|  |  | Control | 4,270 | 1,973 (493.2 ± 78.2) | 46.21 |  |  | 7,198 | 2,348 (587.0 ± 90.2) | 32.62 |  |

SB: small artificial breeding site (6.5 x 9 cm); OV: regular size ovitrap (9 x 12.5 cm); MB: medium artificial breeding site (20.5 x 20 cm); LB: large artificial breeding site (28 x 26 cm).

*All live *Aedes aegypti* larvae, pupae and adults (represented by pupal exuviae) after 24 days were used to estimate the breeding productivity.
